# A Glycolysis-Related Five-Gene Signature Predicts Biochemical Recurrence-Free Survival in Patients With Prostate Adenocarcinoma

**DOI:** 10.3389/fonc.2021.625452

**Published:** 2021-04-19

**Authors:** Zijun Xu, Lijuan Xu, Liping Liu, Hai Li, Jiewen Jin, Miaoguan Peng, Yanrui Huang, Haipeng Xiao, Yanbing Li, Hongyu Guan

**Affiliations:** ^1^ Department of Endocrinology and Diabetes Center, The First Affiliated Hospital of Sun Yat-sen University, Guangzhou, China; ^2^ National Clinical Research Center for Respiratory Disease, The First Affiliated Hospital of Guangzhou Medical University, Guangzhou, China; ^3^ The Translational Medicine Laboratory, The First Affiliated Hospital of Guangzhou Medical University, Guangzhou, China

**Keywords:** prostate adenocarcinoma, biochemical recurrence, glycolysis, gene signature, The Cancer Genome Atlas

## Abstract

Prostate cancer (PCa) is one of the most frequently diagnosed cancers in males worldwide. Approximately 25% of all patients experience biochemical recurrence (BCR) after radical prostatectomy (RP) and BCR indicates increased risk for metastasis and castration resistance. PCa patients with highly glycolytic tumors have a worse prognosis. Thus, this study aimed to explore glycolysis-based predictive biomarkers for BCR. Expression data and clinical information of PCa samples were retrieved from three publicly available datasets. One from The Cancer Genome Atlas (TCGA) dataset was used as the training cohort, and two from the Gene Expression Omnibus (GEO) dataset (GSE54460 and GSE70769) were used as validation cohorts. Using the training cohort, univariate Cox regression survival analysis, robust likelihood-based survival model, and stepwise multiply Cox analysis were sequentially applied to explore predictive glycolysis-related candidates. A five-gene risk score was then constructed based on the Cox coefficient as the following: (−0.8367*GYS2) + (0.3448*STMN1) + (0.3595*PPFIA4) + (−0.1940*KDELR3) + (0.4779*ABCB6). Receiver operating characteristic curve (ROC) analysis was used to identify the optimal cut-off point, and patients were divided into low risk and high risk groups. Kaplan–Meier analysis revealed that high risk group had significantly shorter BCR free survival time as compared with that in low risk group in training and validation cohorts. In conclusion, our data support the glycolysis-based five-gene signature as a novel and robust signature for predicting BCR of PCa patients.

## Introduction

Prostate cancer (PCa) is one of the most frequently diagnosed solid malignancies in men and has become the fifth leading cause of male cancer death worldwide (1, 2). Due to the widespread use of prostate-specific antigen (PSA) serum test and the improvement of overall longevity, the incidence of this disease is increasing. Although radical prostatectomy (RP) leads to a favorable rate of cancer control, approximately 25% of all patients experience biochemical recurrence (BCR), which is determined by rising of serum PSA levels within 10 years of RP (3, 4). BCR indicates increased risk for metastasis and castration resistant PCa (5, 6). Exploring gene expressions that are closely correlated with BCR is of great importance. In this regard, more informative markers for assessing increased risk of BCR are highly needed.

Reprogramming of energy metabolism, especially abnormal activation of glycolysis (also known as the Warburg effect) in the presence of oxygen, has been recognized as one of the central hallmarks of cancer (7, 8). Cancer cells exhibit a higher level of glucose consumption and consequent lactate production (9). Glycolysis facilitates conversion of nutrient uptake into biomass and thereby sustains the rapid cancer cell growth (10). Moreover, activation of glycolysis coupled to increased production of lactic acid, promotes multiple cancer-promoting processes, including tumor invasion and metastasis, angiogenesis, suppression of the local anticancer immune response, as well as hypoxia resistance (11, 12). It has been reported that PCa cells develop high rates of glucose consumption in the metastatic stage (13, 14). PCa patients with highly glycolytic tumors have a worse prognosis (15). However, the association between the glycolysis-related gene signature and biochemical recurrence of PCa is largely unknown. We hypothesized that a signature based on glycolysis-related genes might be a concise and practical tool for predicting the BCR of PCa patients after RP. Here, we develop a glycolysis-based five-gene signature for BCR after RP using large-scale gene expression data obtained from The Cancer Genome Atlas (TCGA) and validate it using two Gene Expression Omnibus (GEO) dataset (GSE54460 and GSE70769).

## Methodology and Methods

All analyses were processed using the R software (v. 3.6.3).

### Data Acquisition

RNA-seq raw counts (Illumina Hiseq 2000v2) and clinical information for TCGA prostate adenocarcinoma (TCGA-PRAD, https://portal.gdc.cancer.gov/) dataset were downloaded using *TCGAbiolinks* (Bioconductor version, Release 3.11) package in R. This study complies with the publication guidelines of TCGA (http://cancergenome.nih.gov/publications/publicationguidelines). The RNA-seq data of 100 patients (GSE54460) and the microarray data of 94 patients (GSE70769) were downloaded from GEO dataset (https://www.ncbi.nlm.nih.gov/gds). We used TCGA dataset as the training set and two GEO datasets as independent validation cohorts. There are 408 patients with complete BCR information in TCGA dataset. Among these, 48 cases had BCR, and 360 cases had no BCR. Among the 100 patients from GSE54460, 97 had complete data and were included in this study. Of these cases, 47 had BCR and 50 had no BCR. Additionally, there are 45 cases with BCR in 92 patients with complete information from GSE70769 (**Table 1**). BCR is defined as two or more consecutively elevated PSA results greater than 0.2 ng/ml.

**Table 1:** Clinical characteristics for study cohorts.

Covariate	Training (n = 408)	Validation I (n = 92)	Validation II(n = 97)
**Age (years)**			
<70	371	NA	89
≥70	37	NA	8
**Pathologic N**			
N0	290	NA	NA
N1	66	NA	NA
Unknown	52		
**Pathologic T**			
≤T2	150	48	81
≥T3	253	42	16
Unknown	5	2	
**RACE**			
White	338	NA	45
Black	46	NA	22
Asian	11	NA	0
Others	1	NA	0
Unknown	12		30
**Surgical margins**			
Negative	NA	50	54
Positive	NA	42	38
Unknown			5
**Gleason score**			
<8	239	75	84
≥8	169	15	13
Unknown		2	
**BCR**			
Yes	360	45	50
No	48	47	47
**Time to BCR (months)**	2.5–64.17	0.36–98.27	0–101.06

### Preliminary Screening of Genes

Since genes with no biologically meaningful expression levels are not of interest in a biological point of view (16), we excluded genes with very low expression level by selecting genes with their expression level above 1 in >50% of the total samples. Moreover, among the patients with the same cancer, different patient experiences totally different clinical outcome. The different mRNA expression levels result in diverse prognostic risks, suggesting that the genes with higher variable expression among different patients are more likely to have predictive values (17, 18). To select genes with variable expressions, we calculated median absolute deviation (MAD), a robust measure of the variability of quantitative data, of every gene, and the genes in the last 20 percent of the total expression variances were excluded.

### Gene Set Selection

A glycolysis-related gene set (HALLMARK_GLYCOLYSIS) containing 200 genes was collected from the Molecular Signatures Database (MsigDB, v7.2).

### Selection of BCR Free Survival-Related Genes

The association between glycolysis-related genes and the BCR of the patients was analyzed in the training cohort. *Survival* package in R was used to perform the univariable cox proportional hazards regression analysis. P < 0.05 was considered as statistically significance, and the gene was selected as a BCR-related gene. We then applied a robust likelihood-based survival modeling approach to further identify BCR-related genes. The analysis was implemented by using the *rbsurv* package in R, and the detailed algorithm is summarized in the previous publication (19).

### Construction and Validation of the Risk Score Formula

Glycolysis-related genes identified in the training set from the previous steps were further weighed by their estimate regression coefficients in the multiply Cox regression analysis and thereby the risk formula was calculated. The risk score for each patient in the training set was calculated with this formula. A receiver operating characteristic curve (ROC) was constructed using the *survival ROC* package in R and the optimal cut-off point was determined with the maximal sensitivity and specificity. ROC figure was plotted by *ggplot2* in R. Based on the cut-off value, the patients were classified into low-risk score and high-risk score groups. “*Survfit*” function in *survival* package was used to plot Kaplan–Meier curves for two distinct groups of patients and to calculate *P* value from log rank test. The association between the five-gene signature and clinical characteristics, as well as the expression levels of five genes in recurrent PCa and non-recurrent PCa were calculated and plotted using function based on *ggbetweenstats* of the *ggstatsplot* R package. The accuracy of the risk score formula was then further validated by fitting in two independent validation cohorts.

## Results

**Figure 1:**
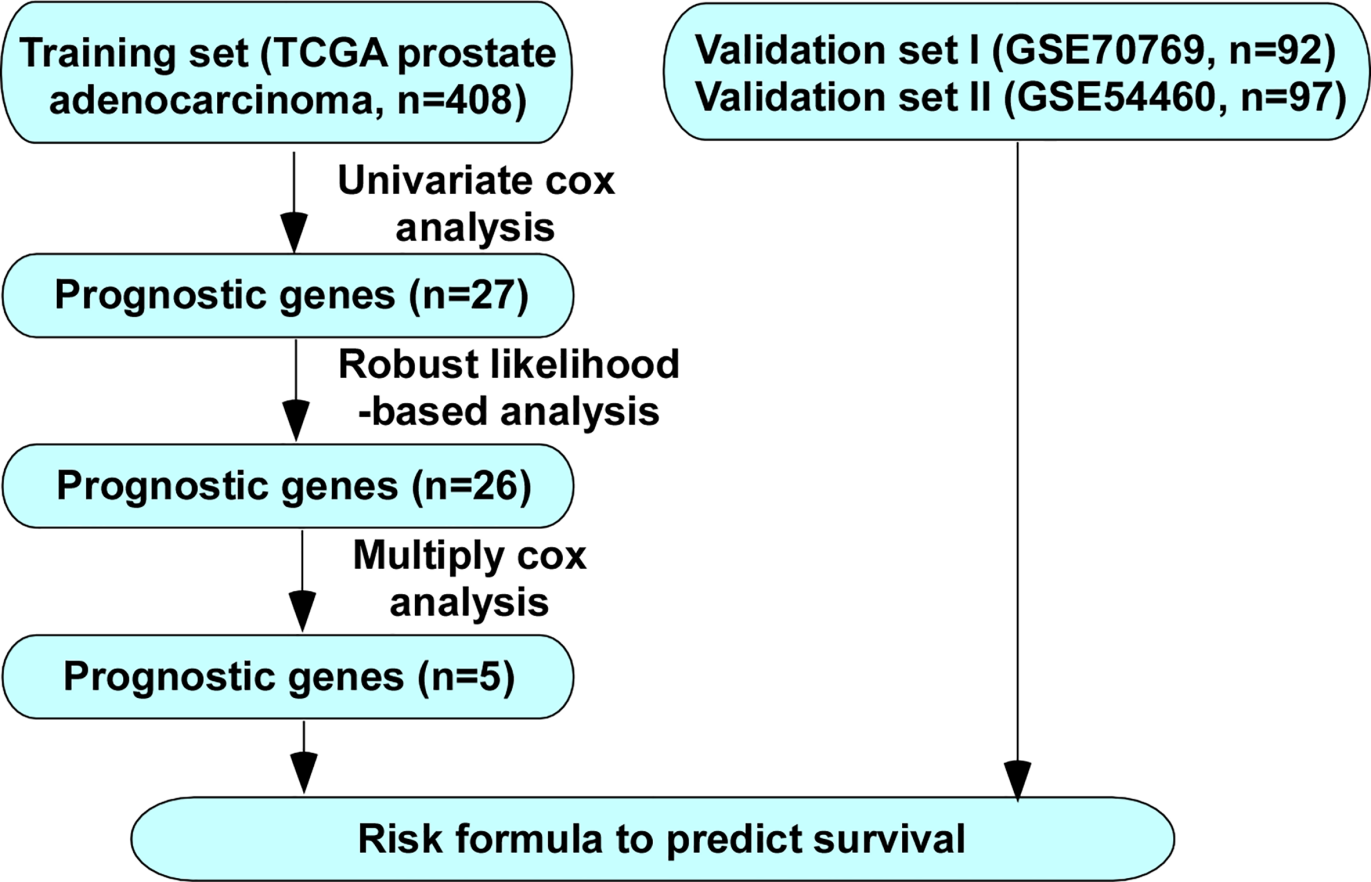
Flow chart of methods for building the five-gene signature for prediction of BCR free survival of PCa patients.

The overall study procedures were summarized in **Figure 1**. Univariate Cox proportional hazard model, robust likelihood-based survival model and multivariate Cox proportional hazards model were used to establish a five-gene signature that can predict the BCR free survival.

### Screening of BCR Free Survival-Related Genes by Univariate Cox Proportional Hazard Modeling

The patients in TCGA with complete BCR information (408 cases) were used as the training set. The 97 patients from GSE54460 and 92 patients from GSE70769 with complete BCR information were used as validation cohorts. The demographic and clinical characteristics of the three cohorts are presented in **Table 1**. A univariate Cox proportional hazards regression model was employed for training cohort using the *coxph* function in the R package *survival* to identify an initial set of 27 BCR free survival-related genes with the threshold of *P* value set as 0.05 (**Table 2**).

**Table 2:** Twenty-seven genes significantly associated with the BCR of patients in the training set (n = 408).

Gene	Hazard Ratio	CI95	P value
CENPA	1.64	1.32–2.05	9.00E-06
KIF20A	1.64	1.31–2.05	1.80E-05
HMMR	1.6	1.26–2.02	1.00E-04
CDK1	1.6	1.26–2.04	0.000114
VCAN	1.48	1.2–1.81	0.000204
AURKA	1.72	1.29–2.29	0.000208
FBP2	1.52	1.21–1.92	0.000415
STMN1	1.74	1.26–2.4	0.000769
DEPDC1	1.35	1.13–1.61	0.000816
COL5A1	1.58	1.19-2.11	0.001765
CHST1	1.72	1.21–2.46	0.002595
PPFIA4	1.36	1.11–1.66	0.002987
IGFBP3	1.59	1.17–2.16	0.003202
GNE	0.68	0.53–0.89	0.004686
KDELR3	0.64	0.47–0.88	0.006206
PAXIP1	2.19	1.22–3.92	0.008217
PYGB	0.53	0.32–0.85	0.009146
AK4	0.68	0.5–0.92	0.012745
ANKZF1	1.82	1.13–2.94	0.013724
TGFBI	1.53	1.07–2.19	0.0198
FUT8	1.85	1.1–3.1	0.020043
ABCB6	1.82	1.03–3.2	0.037675
GYS2	0.52	0.28–0.96	0.037812
TFF3	0.88	0.78–0.99	0.038472
ALDH9A1	0.55	0.3–0.98	0.041664
ANG	0.8	0.64–0.99	0.04322
SLC25A13	1.76	1.01–3.08	0.045132

### Establishment of a Glycolysis-Based Five-Gene Signature by Robust Likelihood-Based Survival and Multivariate Cox Proportional Hazards Modeling

**Figure 2:**
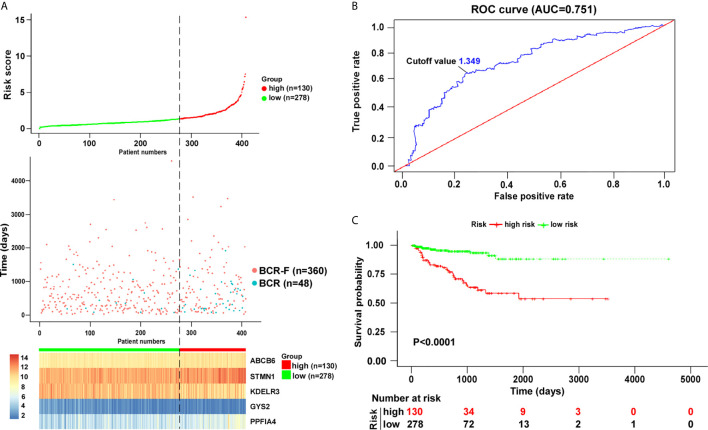
Risk score analysis based on five-gene signature in training cohort. **(A)** Distribution of five-gene-based risk scores, BCR free survival status, and gene expression levels of patients in training cohort. **(B)** ROC curve analyses based on the five-gene signature. **(C)** Kaplan–Meier curves of BCR free survival according to the five-gene signature. P value was calculated with the logrank test.

To increase the feasibility and reliability, we performed the robust likelihood-based survival analysis using the R package *rbsurv*. As shown in **Table 3**, 26 genes were selected using *rbsurv*. Next, we subjected these genes to multivariable Cox analysis using the training set, and each gene’s regression coefficient was generated. This procedure constructed a prediction model containing five genes: glycogen synthase 2 (GYS2), stathmin 1 (STMN1), PTPRF interacting protein alpha 4 (PPFIA4), KDEL endoplasmic reticulum protein retention receptor 3 (KDELR3), and ATP binding cassette subfamily B member 6 (ABCB6). The risk formula used to calculate the risk score was as follows: risk score = (−0.8367*GYS2) + (0.3448*STMN1) + (0.3595*PPFIA4) + (−0.1940*KDELR3) + (0.4779*ABCB6). We then calculated the risk score of each patient in training set using the risk formula. As shown in **Figure 2A**, we visualized risk score distribution and the dash line was used to determine the boundary between high risk group and low risk group. An optimal cut-off was determined based on the ROC analysis (**Figure 2B**). As shown in **Figure 2B**, we selected the point with the maximal sensitivity and specificity as the cut-off point (value = 1.349) and the patients in training set were divided into two groups, high risk group (n = 130) and low risk group (n = 278) (**Figure 2A**). The area under the ROC Curves (AUC) was 0.751 (**Figure 2B**). We evaluated the BCR free survival using Kaplan-Meier method and log-rank test, and the results showed that BCR free survival time of the high risk group was significantly shorter than the low risk group (P <0.0001) (**Figure 2C**).

**Table 3:** Prognosis related genes signature screened using forward selection in the training set (n = 408).

Gene	nloglik	AIC
TGFBI	253.77	509.54 *
TFF3	252.47	508.95 *
IGFBP3	250.92	507.85 *
FUT8	250.22	508.44 *
SLC25A13	248.44	506.88 *
COL5A1	247.83	507.67 *
CHST1	247.32	508.65 *
VCAN	246.72	509.43 *
ANG	246.36	510.73 *
ALDH9A1	245.29	510.57 *
GYS2	241.61	505.23 *
AK4	239.84	503.67 *
PYGB	239.61	505.23 *
GNE	239.34	506.68 *
STMN1	238.92	507.85 *
AURKA	238.44	508.88 *
DEPDC1	237.56	509.13 *
HMMR	237.16	510.31 *
KIF20A	236.94	511.87 *
CDK1	236.71	513.42 *
PAXIP1	236.67	515.33 *
PPFIA4	234.67	513.34 *
CENPA	234.09	514.17 *
KDELR3	231.55	511.10 *
FBP2	230.29	510.57 *
ABCB6	224.03	500.07 *

*Selected.

**Figure 3:**
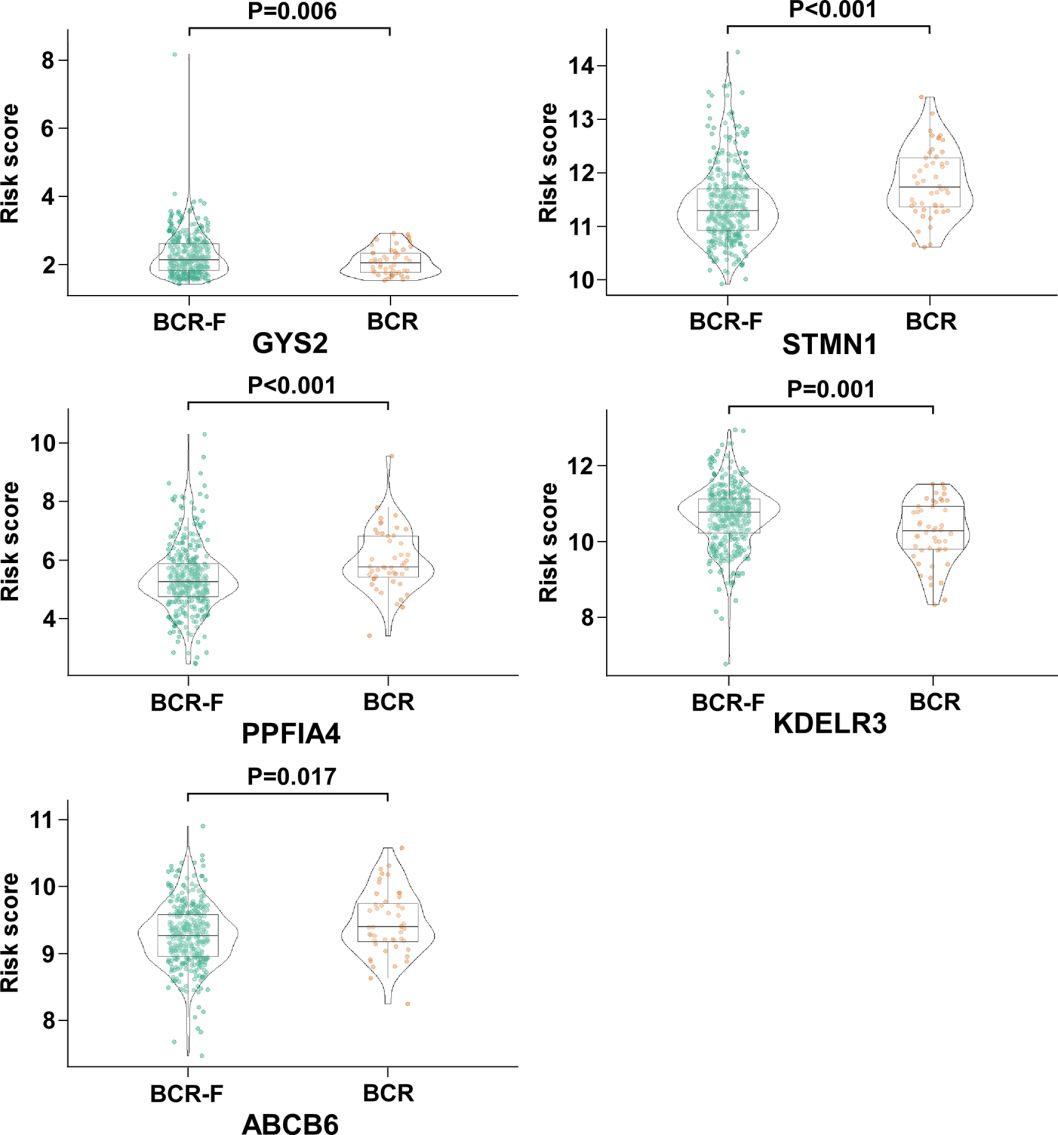
The expression of GYS2, KDELR3, STMN1, PPFIA4, and ABCB6 in BCR and BCR free patients. GYS2 and KDELR3 were highly expressed in BCR free patients. In contrast, the expression levels of STMN1, PPFIA4, and ABCB6 were increased in BCR patients.

Among the five genes, GYS2 and KDELR3 have negative coefficients and were highly expressed in BCR free patients as compared with that in BCR patients. In contrast, the levels of genes with positive coefficients (STMN1, PPFIA4, and ABCB6) were increased in BCR patients (**Figure 3**).

### The Relationship Between Clinical Characteristics and BCR Free Survival-Related Prediction Model

**Figure 4:**
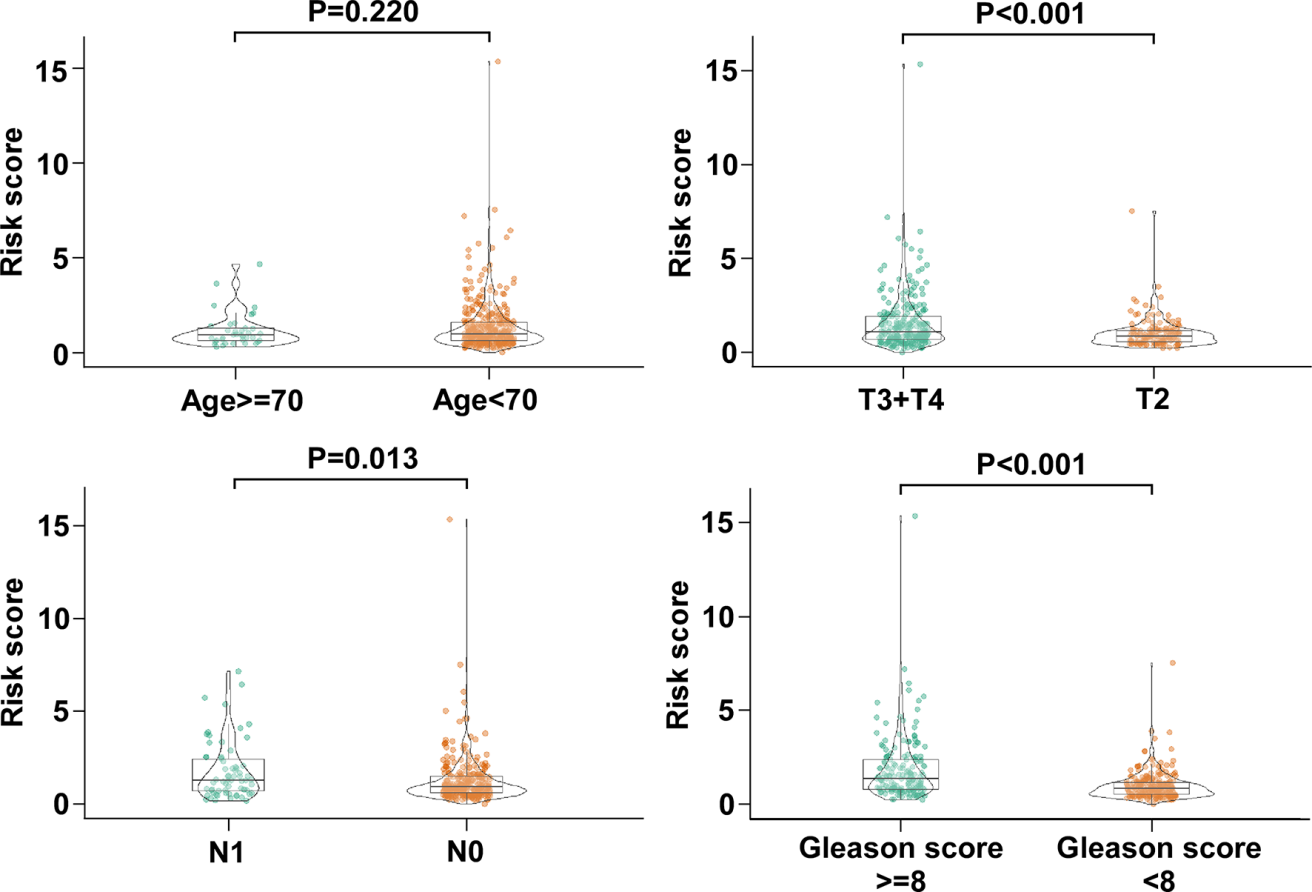
The association between the five-gene signature and clinical characteristics of PCa patients. The distribution of risk scores was associated with pathologic T, pathologic N, and Gleason score, but not age.

Next, we analyzed the association between the risk score value and clinical characteristics of the PCa patients. As shown in **Figure 4**, the risk score value was higher in T3–4 than in T2 (P < 0.001), higher in N1 stage than N0 stage (P = 0.013), and higher in Gleason score ≥ 8 than <7 (P < 0.001). No significance was observed in age <70 and age ≥70 (**Figure 4**).

### Screening of BCR Free Survival-Related Clinical Characteristics

**Figure 5:**
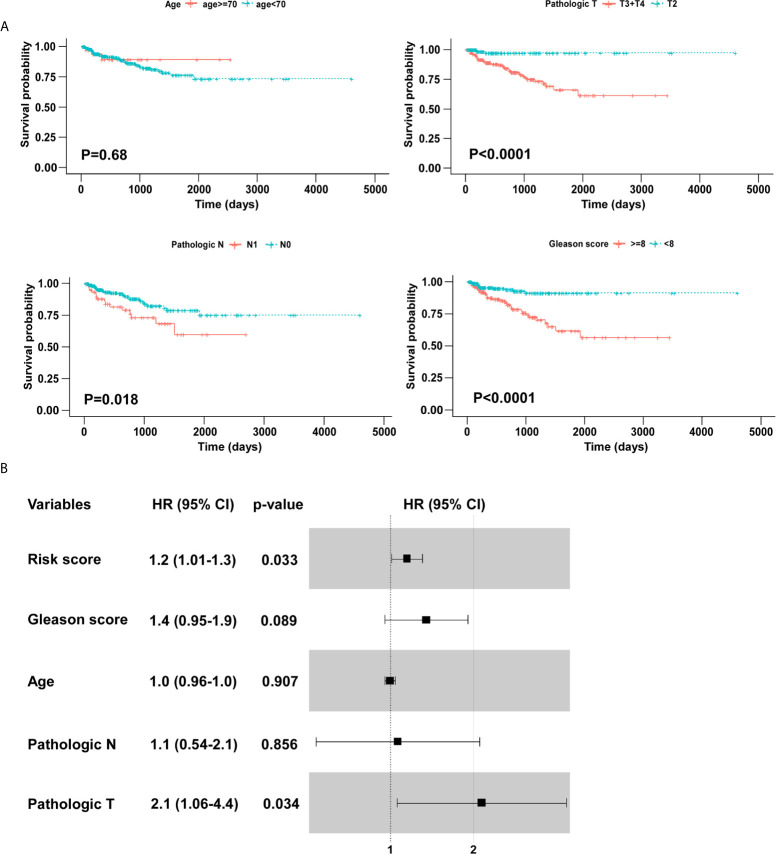
Identification of BCR free survival related clinical characteristics using Kaplan–Meier analyses. **(A)** Pathologic T and Gleason score were significantly associated with BCR free survival. **(B)** The forest plot of risk score and clinical parameters.

We screened the BCR free survival-related clinical characteristics by performing Kaplan–Meier analyses in the training cohort. As shown in **Figure 5A**, pathological T, pathological N, and Gleason score were significantly associated with the BCR free survival of PCa patients. Moreover, a forest plot was constructed using a multivariable Cox regression analysis to visualize the distribution of clinicopathological parameters, including age, pathological T, pathological N, Gleason score and risk score. As shown in **Figure 5B**, the identified five-gene signature was an independent factor affecting BCR free survival in the training cohort.

### Validation of the Five-Gene Signature for BCR Free Survival Prediction

**Figure 6:**
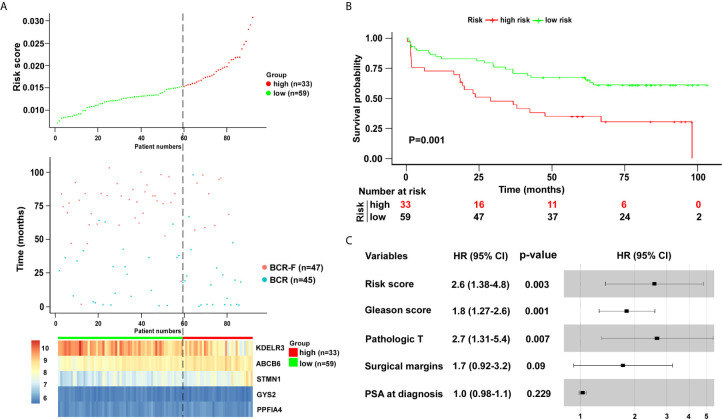
Verification of five-gene signature in validation cohort (GSE70769). **(A)** Distribution of five-gene-based risk scores, BCR free survival status, and gene expression levels of patients in validation cohort. **(B)** Kaplan–Meier curves of BCR free survival according to the five-gene signature. P value was calculated with the logrank test. **(C)** The forest plot of risk score and clinical parameters.

**Figure 7:**
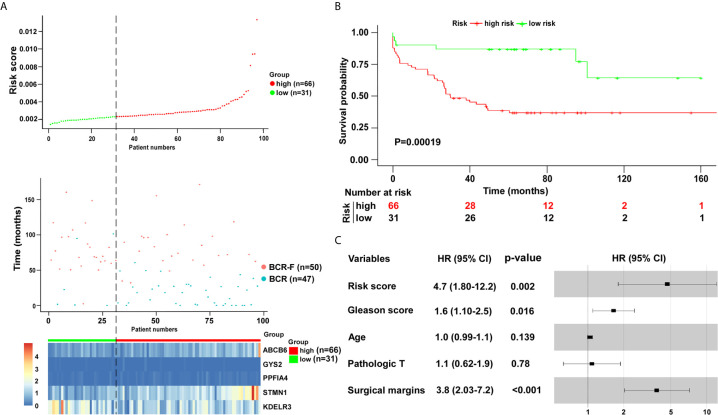
Verification of five-gene signature in validation cohort (GSE54460). **(A)** Distribution of five-gene-based risk scores, BCR free survival status, and gene expression levels of patients in validation cohort. **(B)** Kaplan–Meier curves of BCR free survival according to the five-gene signature. P value was calculated with the logrank test. **(C)** The forest plot of risk score and clinical parameters.

To validate the five-gene signature for BCR free survival prediction, we applied the same analyses to the other two independent validation cohorts, respectively. The distributions of risk scores, BCR status and genes expression were presented in **Figures 6A** and **7A**. Moreover, the results of Kaplan–Meier analysis in two validation cohorts revealed that the BCR free survival time in patients with low risk was significantly longer than that of the patients with high risk (GSE70769: P = 0.001; GSE54460: P = 0.00019) (**Figures 6B** and **7B**). Of note, in consistent with the result in the training cohort, the risk score was also an independent risk factor in two validation cohorts (GSE70769: HR = 2.6, P = 0.003; GSE54460: HR = 4.7, P = 0.002) (**Figures 6C** and **7C**).

## Discussion

Considering that one of the major turning points in PCa development is the progression to BCR, it is highly desirable to effectively assess PCa patients with increased risk of BCR (20, 21). Accumulating researches have been made to identify biomarkers to improve the prediction of patients with BCR. It has been demonstrated that certain clinical parameters, including pathologic stage, Gleason score, lymphonode metastasis, and lymphovascular invasion, are associated with BCR (22–24). Moreover, several gene signatures have been established to predict BCR after prostatectomy. For instance, it has been shown that the cell cycle progression (CCP) score, an RNA expression signature based on the levels of 31 CCP genes, can predict BCR free survival (25). Jiang et al. extracted 696 differentially expressed genes from the TCGA dataset and developed a 15-gene signature (SigMuc1NW) using Elasticnet for prediction of BCR (26). In addition, signatures based on non-coding RNA, such as long non-coding RNAs (lncRNAs) and microRNAs (miRNAs), also have been established to predict BCR. Using lncRNAs that were differentially expressed between tumor and normal prostate tissues, as well as between high and low Gleason score tumor tissues, Shao et al. constructed a seven-lncRNA signature that can predict BCR (27). Moreover, five miRNAs (miR-30c-5p/31-5p/141-3p/148a-3p/miR-221-3p) were identified as independent prognostic biomarkers for BCR (28). However, the predictive value of glycolysis-related gene signature in BCR remains largely unknown. Given the important roles of elevated glycolysis in cancer development and progression, we would like to explore whether it is possible to establish a robust glycolysis-based gene signature to predict the BCR in PCa patients.

As compared with a single biomarker, integrating multiple biomarkers into an aggregated model with bioinformatics analysis would substantially improve the predictive performance (29, 30). Here, we performed a multistep analysis to identify a glycolysis-based gene signature which could predict BCR free survival in patients with PCa. Considering that differentially expressed genes between normal tissues and malignant specimens may not be associated with BCR at all, a univariable Cox analysis was carried out for the primary screening. Robust likelihood-based survival modeling, which selects predictive factors based on the partial likelihood of the Cox model, is commonly used in construction of predictive signature for cancers (31, 32). We used robust likelihood-based survival analysis and multiply Cox regression model to establish a five-gene signature with prediction value. Cut-off scores are often set arbitrarily and vary between reports. ROC curve analysis can be used as an alternative method in the selection and validation of cut-off scores for determining clinically relevant threshold (33, 34). We used ROC analysis to identify the optimal cut-off point for dividing patients into low risk and high risk groups. We found that the BCR free survival time of high risk group was significantly shortened. Importantly, results from the validation cohorts confirmed the robustness of the glycolysis-based five-gene signature, suggesting the excellent performance and consistency of this model throughout the training cohort and two validation cohorts. These data indicate that the five-gene signature exhibits a robust prediction value for BCR free survival in patients with PCa.

We identified five glycolysis-related mRNAs (GYS2, STMN1, PPFIA4, KDELR3 and ABCB6) which were associated with BCR after RP. Among the 5 genes, the expression levels of the genes with negative coefficients (GYS2 and KDELR3) were increased in BCR free patients. Moreover, STMN1, PPFIA4, and ABCB6 have positive coefficients, and their expression levels were upregulated in patients with BCR. It is of note that bulk tumor mass is composed of diverse cells, including malignant, stromal and immune cells (35). Moreover, the datasets in current study are based on the conventional bulk-level molecular profiling. The molecular profiles of these approaches represent an average readout from all cell types within the tissue. The averaging over the individual cells leads to information loss (36). Therefore, the change of the expression levels of the five genes may partially be due to the alterations in stromal amount. Recent developments in single-cell RNA sequencing (scRNA-seq) have enabled the transcriptomes of single cells to be sequenced in a high throughput manner (37). ScRNA-seq provides a comprehensive and precise analysis of the cancer cell genome (38). Further studies based on the new powerful approach in large cohort of PCa patients will provide new insights into the characteristics of the disease and facilitate the exploration of new markers and therapeutic targets.

Some of the 5 genes have been implicated to be involved in cancers, including PCa. GYS2 encodes a protein that catalyzes the rate-limiting step in the synthesis of glycogen (39). It has been reported that GYS2 acted as a tumor suppressor in hepatocellular carcinoma (HCC) (40). The research found that GYS2 inhibited the proliferation of HCC cells *via* a negative feedback loop with p53 (40). A series studies have demonstrated the oncogenic role of STMN1 in various kinds of cancers (41–43). STMN1 was overexpressed in PCa and its expression was associated with the malignant behavior of the disease (44). MiR-34a, a tumor suppressor miRNA, inhibited the progressive phenotypes of PCa cells *via* directly regulating STMN1 (45). PPFIA4 belongs to the liprin-alpha gene family and inhibition of PPFIA4 reduced pancreatic cancer cell proliferation and invasion (46). Moreover, suppression of PPFIA4 promoted chemosensitivity of small lung cancer (SCL) cells under hypoxia (47). KDELR3 is a gene which encodes a member of the KDEL endoplasmic reticulum protein retention receptor family. The research by Marie et al. showed that silencing of KDELR3 reduced lung colonization of melanoma cells in experimental metastasis assays *via* regulating the metastasis suppressor, KAI1 (48). In addition, overexpression of ABCB6, a member of the ATP-binding cassette (ABC) transporter superfamily, could enhance the accumulation of protoporphyrin IX and improve the efficacy of 5-aminolevulinic acid-based photodynamic therapy in glioma (49). Karatas et al. reported that the expression of ABCB6 was elevated in PCa tissues as compared with that in normal prostate tissues. Consistently, they also found that ABCB6 was overexpressed in recurrent PCa in comparison with non-recurrent PCa (50). However, the unrecognized roles of GYS2, PPFIA4 and KDELR3 in PCa are worth further investigating to identify the biological functions and underlying mechanisms of theirs in the development and progression of the disease. Further delineation of molecules from the signature will provide new insights into the etiology of the disease and might uncover potential therapeutic targets.

In conclusion, we conducted an integrated study to develop a glycolysis-based five-gene signature for the prediction of the BCR free survival of PCa patients after RP. Future prospective clinical trials are warranted to evaluate the clinical utilization of this signature.

## Data Availability Statement

Publicly available datasets were analyzed in this study. This data can be found here: https://portal.gdc.cancer.gov/, The Cancer Genome Atlas prostate adenocarcinoma dataset.

## Author Contributions

ZX, LX, and LL: data acquisition, analysis, and manuscript writing. HL, JJ, MP, and YH: interpretation of data and statistical analyses. HX, HG, and YL: study concept, design, and supervision. All authors contributed to the article and approved the submitted version.

## Funding

The funding for this project was provided by the National Natural Science Foundation of China (No. 82073050 and 81772486); Guangdong Basic and Applied Basic Research Foundation (No. 2019A1515012046 and 2020A1515010049); Guangdong Medical Scientific Research Foundation (No. A2020343); and Young Teachers Cultivate Projects of Sun Yat-sen University (No. 20ykpy60).

## Conflict of Interest

The authors declare that the research was conducted in the absence of any commercial or financial relationships that could be construed as a potential conflict of interest.
